# Entomopathogenic fungi decrease *Rhizoctonia* disease in potato in field conditions

**DOI:** 10.7717/peerj.9895

**Published:** 2020-09-16

**Authors:** Oksana G. Tomilova, Elena M. Shaldyaeva, Natalia A. Kryukova, Yulia V. Pilipova, Natalia S. Schmidt, Viktor P. Danilov, Vadim Y. Kryukov, Viktor V. Glupov

**Affiliations:** 1 Institute of Systematics and Ecology of Animals SB RAS, Novosibirsk, Russia; 2 Department of Plant Protection, Novosibirsk State Agrarian University, Novosibirsk, Russia; 3 Federal Scientific Centre of Agro-BioTechnologies (SFSCA) of the RAS, Novosibirsk, Russia

**Keywords:** *Rhizoctonia solani*, *Metarhizium robertsii*, *Beauveria bassiana*, Endophyte, Antagonism, Peroxidase, Biocontrol, Siberia, Yield

## Abstract

*Rhizoctonia* potato disease is widespread in the world and causes substantial yield and quality losses in potato. This study aimed to evaluate the efficacy of entomopathogenic fungi *Metarhizium robertsii* and *Beauveria bassiana* in the inhibition of potato *Rhizoctonia* complex disease. The efficacy of the entomopathogenic fungi *M. robertsii* and *B. bassiana* in the defense of potato against *Rhizoctonia* disease (stem cancer, black scrulf and other forms of manifestation on tubers) was estimated under field conditions in Western Siberia. Preplanting treatment of the tubers with *B. bassiana* decreased *Rhizoctonia* disease in the stems and stolons. At the same time, treatment with *M. robertsii* did not cause a decrease in *Rhizoctonia* disease in these organs. However, both fungi decreased the sclerotium index on the tubers of new crops. We demonstrated two mechanisms of inhibition of *Rhizoctonia solani* by *M. robertsii* and *B. bassiana*, including (1) direct effect, expressed as inhibition of *R. solani* sclerotium formation in cocultivation assays, and (2) indirect effect, which is associated with increased peroxidase activity in potato roots under the influence of colonization by entomopathogenic fungi. We suggest that the treatment of seed tubers with *B. basiana* can effectively manage *Rhizoctonia* disease during the plant vegetative season and that both fungi significantly improve the quality of the new tuber crop.

## Introduction

*Rhizoctonia solani* complex disease is a widespread destructive potato disease that occurs in all potato-growing countries (Baker, 1970; Carling, Leiner & Westphale, 1989; Wilson et al., 2008a). The decrease in potato production resulting from this disease reach 30% (Tsror, 2010). *Rhizoctonia* disease develops epiphytotically, producing serious lesions on plants every year in Western Siberia and especially in years with stressful conditions (Shaldyaeva & Pilipova, 1996). The disease manifests in several forms, affecting sprouts, stems, stolons and tubers. In the underground organs, the disease manifests as dry ulcer rot. The injuriousness of the disease is primarily associated with its effects on young potato sprouts and stems, which lead to the die-out of sprouts, sparseness and weakening of plants, and a reduction in tuber numbers (Carling, Leiner & Westphale, 1989). The damage caused to potatoes by *Rhizoctonia* disease under Siberian continental climatic conditions can reach 67–70.8% with a disease prevalence of 80–100% (Shaldyaeva & Pilipova, 2017). In potato tubers, the disease manifests as black scrulf, dry core and scab-like symptoms (Baker, 1970). In Siberia, all of these symptoms are observed on potato tubers (Shaldyaeva & Pilipova, 1996).

The protection of potato from *R. solani* involves procedures aimed at both suppressing seed infections and rehabilitating soil. The use of microorganisms that colonize the roots and aboveground organs of plants leads to the inhibition of phytopathogens. For example, the high biocontrol potential of *Pseudomonas* and *Bacillus* spp. bacteria, arbuscular mycorrhizal fungi and endophytic fungi can decrease plant damage caused by pathogens and stimulate plant growth (Wilson et al., 2008b; Weselowski et al., 2016; Adam et al., 2016; Schreiter et al., 2018; Bagy et al., 2019; Jiang et al., 2019).

Entomopathogenic fungi from genera *Metarhizium* and *Baeuveria* may engage in mutualistic interactions with plants and suppress the infections caused by fungal and viral phytopathogens (Moonjely, Barelli & Bidochka, 2016; Jaber & Enkerli, 2017; Jaber & Ownley, 2018; Vega, 2018; Bamisile et al., 2018; Branine, Bazzicalupo & Branco, 2019). The suppression of phytopathogenic fungi after the treatment of plants with different species of *Beauveria* and *Metarhizium* fungi has been shown in the following systems: *Pythium myriotylum* and *Rhizoctonia solani*—tomato and cotton plants (Ownley et al., 2008), *Fusarium oxysporum*—onion (Flori & Roberti, 1993), powdery mildew—cucumber (Kim, Goettel & Gillespie, 2010), yellow mosaic virus—pumpkin (Jaber & Salem, 2014), and downy mildew—grape (Jaber, 2015). The hypotheses put forth to explain the suppression of fungal diseases include the following: competition between endophytes and phytopathogens for a substrate (Ownley et al., 2004; Ownley, Dee & Gwinn, 2008); antagonism and synthesis by endophytes that protect plant secondary metabolites (Collemare et al., 2014; Ríos-Moreno et al., 2016; Garrido-Jurado et al., 2017); and activation of protective plant systems as a result of low-level stress (Ownley, Gwinn & Vega, 2010; Maksimov et al., 2015). However, the mechanisms of this suppression are still not well understood.

The generation of reactive oxygen species on the cell surface is one of the early responses to abiotic and biotic stress (Bolwell, 1999; Van Doorslaer et al., 1999). Superoxide dismutase, peroxidase and catalase are the most important high-molecular-weight plant antioxidants (Alscher, Donahue & Cramer, 1997). In particular, peroxidase participates in the formation and lignification of plant cell walls (lignin and suberin formation), ethylene biosynthesis, the regulation of auxin levels, and tissue protection against different damages and infections caused by pathogenic microorganisms (Wiesel et al., 2014). In addition, this enzyme plays an important role in plant resistance against abiotic stresses (Gaspar et al., 1991; Zhang & Kirkham, 1994; Yang, Cheng & Liu, 2007; Muratova et al., 2015). The level of peroxidase activity depends on the different external and internal influences; therefore, many authors use the assessment of peroxidase activity as a test for the determination of plant immune status during colonization by endophytic fungi and bacteria (Hirano et al., 2008; Senthilraja et al., 2013; Maksimov et al., 2014; Maksimov et al., 2015).

The decrease in plant injury by phytopathogens after treatment with entomopathogenic fungi is likely caused by a combination of different mechanisms depending on both the environmental conditions and the studied species. It is important to note that most studies of the interactions between endophytic entomopathogenic fungi, plants and phytopathogens have been conducted in laboratory conditions. However, the results obtained from laboratory assays are not always consistent with the results obtained from field experiments due to the influence of a large number of environmental factors. There have been no studies on the effect of entomopathogenic fungi on the interaction between potato and *Rhizoctonia* in field conditions. The present investigation was focused on the interactions of *M. robertsii* and *B. bassiana* with *R. solani*. The study was performed under both in vitro and Western Siberian field conditions in potato plants subjected to the preplanting treatment of seed tubers with *M. robertsii* and *B. bassiana* conidia. The damage to the plants caused by *Rhizoctonia* disease, plant growth and peroxidase activity were estimated during the growing season. In addition, the crop characteristics of the potatoes treated with the fungi were analyzed.

## Materials & Methods

### Fungal isolates

We used isolates of the entomopathogenic fungi *M. robertsii* (isolate P-72) and *B. bassiana* (isolate Sar-31) from the collection of microorganisms of the Institute of Systematics and Ecology of Animals (Siberian Branch of the Russian Academy of Science). The fungal species were identified on the basis of the sequence of the translation elongation factor (EF1*α*) region (Kryukov et al., 2017). To prepare conidia for the field experiments, the fungi were cultivated on twice-autoclaved millet and then sifted through a 1 mm sieve. Conidium concentrations were determined with a Neubauer hemocytometer. *R. solani* (anastomose group—AG3) from the laboratory of the Department of Plant Protection (Novosibirsk Agricultural State University) was used in the work. The fungus was isolated from infected potato tubers in Western Siberia.

### Antagonistic action of *M. robertsii* and *B. bassiana* toward *R. solani*

The antagonistic interactions between the entomopathogenic fungi (*M. robertsii* and *B. bassiana*) and *R. solani* were estimated using the dual culture method on potato dextrose agar (PDA) in the 90 mm Petri dishes (Sobowale et al., 2010). Ten-millimeter mycelium plugs from 5-day-old cultures of *R. solani* and one of the antagonists (*M. robertsii* or *B. bassiana*) were placed in the Petri dishes 3 cm from each other. *M. robertsii* or *B. bassiana* were plated two days before *R. solani* due to high growth rate of *R. solani* growth. Dishes inoculated with *R. solani* alone were used as controls. The plates were incubated at 25 °C for 20 d in darkness, and the interaction types between the entomopathogenic fungi and *R. solani* were estimated at 5, 10, 15, and 20 d. The differences between the diameter of the *M. robertsii* or *B. bassiana* colonia growth (10, 15, 20d) and diameter at the cultures contact time (5d) was used for estimation of the entry of entomopathogenic fungi into the colony of *R. solani.* Interactions between mycelia in the contact area of fungi were observed by light microscopy (Axioskop 40, Carl Zeiss, Germany) and electron microscopy (Hitachi TM-1000, Japan). Each treatment included five replicates, and the entire experiment was conducted twice.

### Field experiment

#### Locality, soil and *Rhizoctonia* infectious background

Field experiments were carried out in 2018 on Priobskoe Farm in the Novosibirsk region of Russia (55°06′N, 82°77′E). The field permit was not required, since all experiments were carried out at the field sites of the Institute of Systematics and Ecology of Animals. The soil type was sod-podzolic. The soil organic matter content was 2.18%, and the pH was 5.1. Previously, white cabbage was cultivated in the field.

The natural soil-borne and seed-borne loads of *R. solani* have been used during the field studies. The count of *R. solani* in the soil of experimental field was determined via the multiple soil tablet method (Henis et al., 1978) using selective medium (Ko & Hora, 1971). The soil population of the pathogen was below the biological threshold of harmfulness: 0.2 propagules per 100 g of soil before planting (May 26, 2018). The seeds of tubers (“Red Scarlet” line) exhibiting high levels of seed-borne infection (black scab, dry core symptoms, scab-like symptoms). The 60% of tubers had black scurf at the time of planting.

#### Treatments and planting

The field experiment included three treatments: (1) *M. robertsii*, (2) *B. bassiana* and (3) the control. The conidia of the fungi were suspended in a water-Tween 20 solution (0.04%) at a concentration of 5 × 10^7^ conidia/mL. Potato tubers were immersed in the suspensions for 30 min immediately before planting. Control tubers were treated with the water-Tween solution alone. The applied volume of the suspensions was 10 L per 50 tubers (the average weight of one tuber was 80-90 g). Tubers were planted according to Dutch technology (https://en.blabto.com/1565-dutch-potato-growing-technology.html) with a distance between plants of 30 × 70 cm. Four plot areas with randomized placement of plants (10 m^2^, 50 plants per plot) were subjected to each treatment. Potatoes were planted on May 28 and harvested on August 24 in 2018.

#### Weather conditions in the growth season

**Figure 1:**
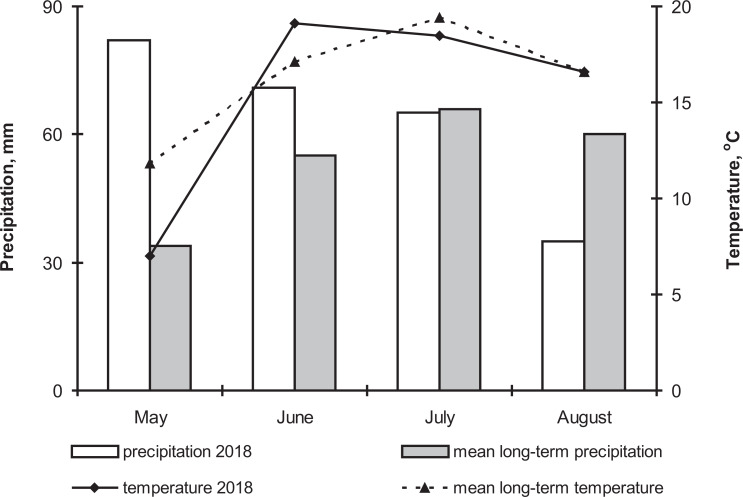
The dynamics of temperature and precipitation by month in the growing season (2018) and comparison with the average long-term data.

The 2018 growth season was characterized by high precipitation (241% of the long-term average) and low temperatures in May (4.8 °C lower than the long-term average) (Fig. 1). These meteorological conditions caused a delay in potato planting and seedling emergence. Moreover, cold temperatures in July caused a delay in the potato flowering and ripening periods. Precipitation and temperature in June were 29% and 2 °C higher compared to the long-term average. Precipitation and temperature in July were close to long-term norms, but August was arid (precipitation was 42% lower compared to long-term average). That is, weather conditions in the first half of the season were appropriate for the active development of both the phytopathogenic and entomopathogenic fungi (Carling & Leiner, 1990; Inglis et al., 2001).

#### Experimental time-points

At 4 and 7 weeks postplanting, we estimated the parameters of *Rhizictonia* infection in potato plants, the growth characteristics of the plants, the colonization of the plants with *Beauveria* and *Metarhizium* and peroxidase (POX) activity in the plants. The CFUs of *Beauveria* and *Metarhizium* in the soil were estimated before planting (May 26) and at the end of the experiment (August 24). Crop parameters were estimated on August 24.

At 4 and 7 weeks postplanting samples were hand-picked and following plants parameters have been estemated: damage of the stems, growth characteristics, colonization with entomopathogenic fungi (*Beauveria* and *Metarhizium*) and POX activity.

### Parameters of *Rhizictonia* infection

*Rhizoctonia* stem cancer development on potato plants was estimated by the Frank method (Frank, Leach & Webb, 1976). We used the following scale gradation: 0 points—no damage sites; 1 point—one damage site with a length of less than 25 mm; 2 points—one damage site with a length close to 26-50 mm or several minor damage sites of less than 50 mm; 3 points—one or more damage sites with a length of more than 50 mm without encircling the stem; 4 points—one or more damage sites with a length of less than 25 mm with damage encircling the stem; 5 points—one or more damage sites longer than 25 mm in length with damage encircling the stem.

The *Rhizoctonia* stem lesion index (*RSI*) was calculated for each plant according to the following generally accepted formula: *RSI* = ∑(*n* × *b*)∕*N* × *H* × 100%, where *n*—number of damaged stems; *b*—corresponding site of damage; *N*—total quantity of the stems; *H*—high point of scale gradation. The percentage of fallen and damaged stolons among the total stolon count was estimated on the same plants. Five plants from each replication were randomly selected and used for analysis.

To determine the infection rate of the seeds and the new crop tubers, samples of these materials were washed (120 samples from each treatment), and, all *Rhizoctonia* disease manifestations were rated in each sample. These manifestations were as follows: single sclerotia, sclerotia occupying up to 10, 25 or 50% of the surface area of the tuber, dry core symptoms, scab-like symptoms and cracks. All disease forms were combined in the sclerotium index (*Si*). The index was estimated according to Jager & Velvis (1983) with minor modifications. We used the upgraded method corrected by considering both the *Rhizoctonia* disease forms and damage levels of the tubers. *Si* was determined with the following formula: *Si* = *hy* + 3.5*l* + 5*m* + 6*h*∕*c* + *hy* + 1 + *m* + *h*, in which *c, hy, l, m,* and *h*—are the weights (kg) of the tubers of the classes; *c*—clean tubers; *hy*—tubers with only hyphae forms (scab-like symptoms, dry core symptoms, cracks); *l*—tubers with a low level of black scab (single sclerotia or sclerotia occupying up to 10% of the tuber’s surface); *m*—tubers with an intermediate level of black scab (sclerotia occupying up to 25% of the tuber’s surface); *h*—tubers with a high level of black scab (sclerotia occupying up to 50% of the tuber’s surface); 3.5, 5, 6—numerical coefficients corresponding to the damage levels.

The biological efficiency (BE) of the entomopathogenic fungi was calculated using the following formula: *BE* = (*R* − *r*)∕*R* × 100%, where *R*—disease lesion index in the control treatment, and *r*—disease lesion index in the experimental treatment.

### Potato plant growth and the tuber crop

Growth parameters were estimated on plants that were used for the analysis of *Rhizoctonia* disease development (5 plants per replicate, 20 plants per treatment). The length of all stems of each plant was measured from the parent tuber to the apex, followed by the calculation of the arithmetic mean. Fresh weight was measured in the field immediately after removal of soil and parent tubers. Weight was determined using a Polaris PKS 0832DG scale with an accuracy of 1 g.

Potato tuber characteristics were analyzed at harvest time according to the experimental treatments (10 plants per replicate, 40 plants per treatment). The total weight of the tubers and their distribution among different weight groups were estimated with an accuracy of 1 g. Three tuber groups were formed according to theirs masses: less than 35 g (small), 36–180 g (medium) and more than 180 g (large).

### Estimation of plant colonization by entomopathogenic fungi and their CFUs in soil

Plant and soil colonization by the entomopathogenic fungi *M. robertsii* and *B. bassiana* was estimated through plating on the modified Saburoad medium (10 g peptone, 40 g D-glucose anhydrous, 20 g agar, 1 g yeast extract). To prevent growth of bacteria and sporophytic fungi, we added an antibiotic cocktail containing 0.35 g/L cetyltrimethylammonium bromide, 0.05 g/L cycloheximide, 0.05 g/L tetracycline, and 0.6 g/L streptomycin to the culture medium. To estimate plant colonization by the entomopathogenic fungi, we examined the roots, stems and leaves (5 plants from each replicate and 20 plants of each treatment). The potato organs were sterilized with 0.5% sodium hypochlorite suspension and 70% ethanol (Posada et al., 2007). The organs were imprinted on the medium surface and then plated on the medium in 90 mm Petri dishes (McKinnon et al., 2017). The samples for which fungal growth appeared in the imprints were excluded from the analysis. The roots of the potato plants were also plated without surface sterilization. The roots were vortexed 3 times (1 min at 180 rpm each time) in a water-Tween 20 solution (0.04%) and plated on the above-mentioned medium in Petri dishes.

To estimate the soil CFUs of the entomopathogenic fungi, we used soil samples from the potato root area (one sample from each replication of 3 root areas combined). The soil samples (5 g) were diluted in 40 mL of a water-Tween solution (0.1%) and vortexed for 10 s, followed by incubation in the shaker for one hour at 180 rpm. A 100 µL aliquot of the soil solution from each sample was plated on the above-mentioned medium in Petri dishes.

The dishes containing the plant and soil samples were incubated at 24 °C for 14 d, followed by the detection of fungal growth from the plant organs and the calculation of CFU counts in the soil samples. The fungi belonging to *Metarhizium* and *Beauveria* were identified by light microscopy.

### Estimation of POX activity

In this work, we used plant organ samples obtained from potato plants that had been planted 4 and 7 weeks earlier. Stems without symptoms of *Rhizoctonia* disease were selected to analyze POX activity. Roots and leaves (five samples in each replication) were washed carefully and separately tested to determine POX activity. For sample preparation, 100 mg samples of leaves or roots were placed in 100 µL of cooled phosphate buffer (PBS; pH 7.4; 0.01M containing 0.15 M NaCl and 1% (w/v) phenylmethylsulfonyl fluoride and 1% dithiothreitol), followed by sonication in an ice bath with three 15 s bursts using an ultrasonic homogenizer (Bandelin electronic GmbH & Co, Berlin, Germany).

The homogenate was centrifuged at 10,000 g for 20 min at 4 °C. The supernatant was used for the measurement of enzyme activity. To measure POX activity, 2.5 µL aliquots of the plant samples (root or leaf supernatant) were added to the reaction mixture containing 100 µL of 1.7 mM H_2_O_2_ (prepared fresh daily) in PBS and 100 µl of 2.5 mM 4-aminoantipyrine with 0.17 M phenol. The reagents were dissolved in deionized water. The absorbance at 510 nm was measured after 10 min for leaves and after 4 min for roots at 25 °C in a 96-well plate reader. Enzymatic activity was measured in units of the optical density (ΔA) of the incubation mixture during the reaction per 1 min and 1 mg of protein. The concentration of protein in the supernatants was determined via the Bradford method (1976). For the calibration curve, bovine serum albumin was used (from 100 µg to 1,000 µg).

### Statistical analyses

Data analyses were performed using Statistica 8 (StatSoft Inc., USA) and PAST 3 (Hammer, Harper & Ryan, 2001). The normality of the data distribution was checked with the Shapiro–Wilk W test. Normally distributed data were analyzed by one-way ANOVA followed by Fisher’s LSD post hoc test. Non-normal data were analyzed with the Kruskal-Wallis test followed by Dunn’s post hoc test. Student’s *t*-test was used to estimate the data of antagonistic activity in dual-plate assays. Fisher’s exact test was used to estimate differences in the fungal colonization of plants.

## Results

### Antagonistic properties of *M. robertsii* and *B. bassiana* toward *R. solani* in vitro

**Figure 2:**
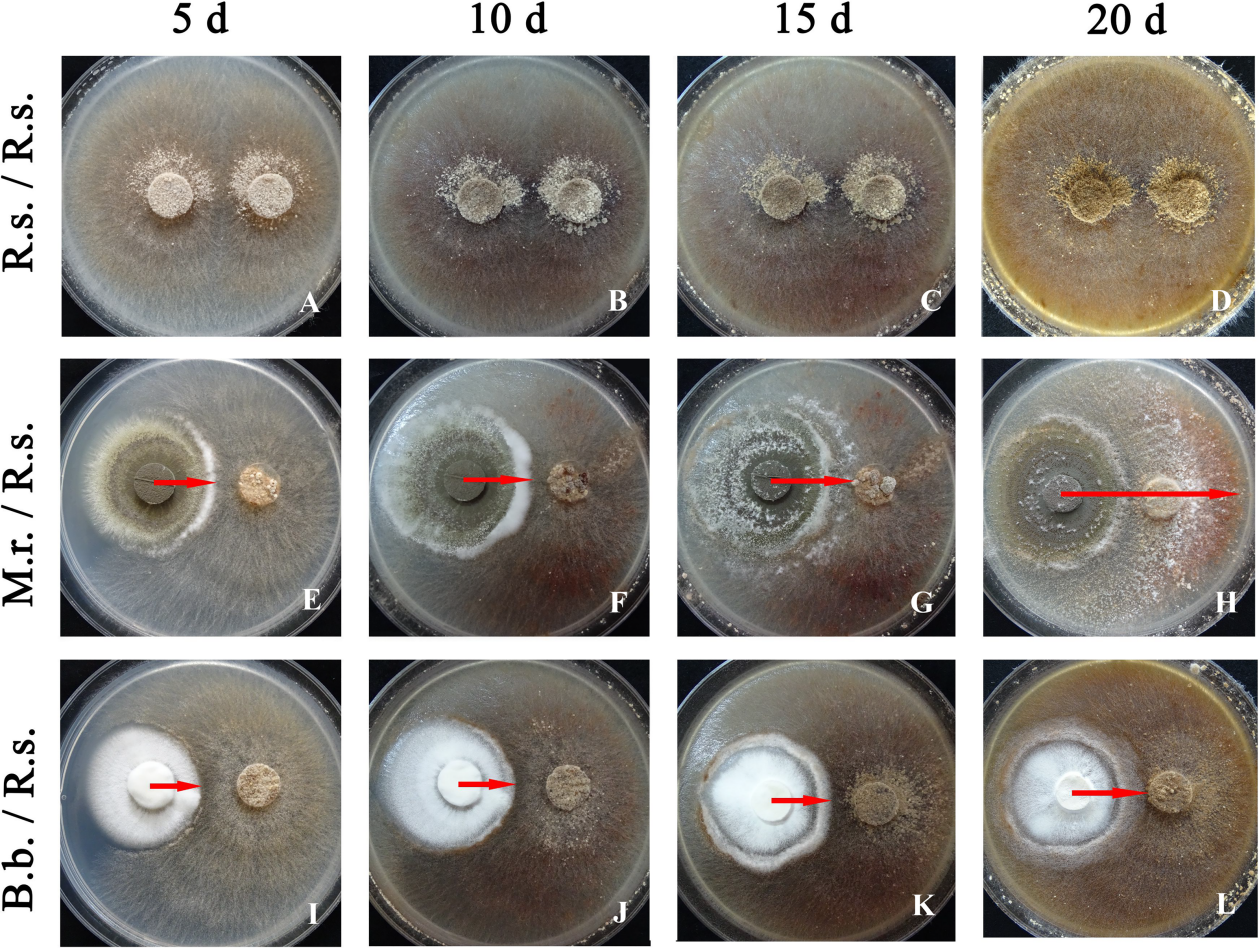
Antagonistic activity of *M. robertsii* (M.r.) and *B. bassiana* (B.b.) against *R. solani* (R.s.) at 5, 10, 15 and 20 d when grown in dual plate cultures on PDA. (A–D) R.s. (control); (E–H) M.r with R.s.; (I–L) B.b. with R.s. Arrows indicate the growth region of an entomopathogenic fungus on the surface of a colony of *R. solani* in dual-plate assays.

The mycelium of the *R. solani* strain was characterized by fast growth. In the Petri dishes containing two plugs with a pure culture of *R. solani* (Fig. 2, R.s./R.s.), almost the entire surface of the medium was occupied after 5 d, and early sclerotia started to form.

**Figure 3:**
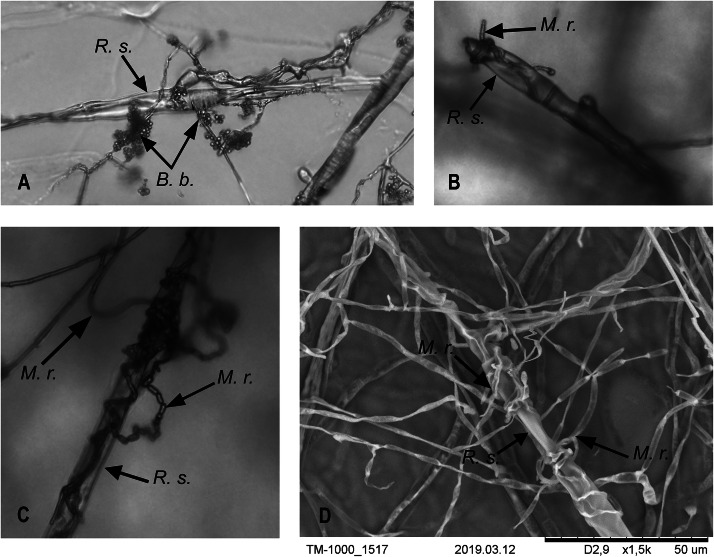
Interaction of *M. robertsii* (M.r.) and *B. bassiana* (B.b.) hyphae with *R. solani* (R.s.). (A–C) Light microscopy × 200; (D) electron microscopy.

We did not observe sterile zones and inhibition of phytopathogen radial growth under the influence of the entomopathogenic fungi. The contact of entomopathogen colonies with *R. solani* occurred after 5 d. However, the morphology of the fungal cultures was altered under joint cultivation. In the case of *M. robertsii*, a sterile (aconidiogenic) zone of the entomopathogenic fungus was formed over a 5 to 10 d of cocultivation (Fig. 2, M.r. / R.s.). The mycelium of *M. robertsii* subsequently began to actively grow on the surface of the *R. solani* mycelium; after 15 d, it reached the phytopathogen plugs, and it spread across the entire surface of the medium in the Petri dish at 20 d. Microscopic observation showed that the mycelium of *R. solani* was actively braided by the *M. robertsii* hyphae and consequently began to thin and dry out (Figs. 3B, 3C and 3D). In the zone where *M. robertsii* was actively growing, the surface mycelium of *R. solani* was significantly degraded. Importantly that *M. robertsii* caused a delay in sclerotium formation of *R. solani*.

*R. solani* growth was also stopped by *B. bassiana.* The *R. solani* mycelium was condensed at the growth border (Fig. 2, B.b./R.s.). *B. bassiana* also began to grow on the *R. solani* mycelium after 10 d of dual cultivation. In contrast to *M. robertsii,* it grew more slowly and densely (*T*-test, *P* < 0.05 between fungi, Table 1) and reached the *R. solani* plugs at 20 d. The microscopic examination showed an interaction that was similar for *M. robertsii* and *R. solani*, with braiding of *R. solani* hyphae by *B. bassiana* (Fig. 3A).

**Table 1:** Antagonistic activity of entomopathogenic fungi against *R. solani* in dual-plate assays.

**Entomopathogenic fungus**	**Entry into the *R. solani* colony, mm (mean ± SE)**		
	**10d**	**15d**	**20d**
*B. bassiana*	1.41 ± 0.14^a^	5.41 ± 0.22^a^	9.65 ± 0.14^a^
*M. robertsii*	1.65 ± 0.22^b^	6.59 ± 0.22^b^	35.53 ± 0.44^b^

**Notes.**

The different letters indicate significant differences between fungi at each time point (*P* < 0.05).

Thus, in the dual growth assays, the *M. robertsii* strain showed stronger antagonistic properties than the *B. bassiana* strain. Both strains led to the necrotization of the aerial mycelium and prevention of full-fledged sclerotium formation in the fungus contact zone.

### *Rhizoctonia* disease development in plants during the vegetation period and in the tubers of a new crop

It was shown that entomopathogenic fungi actively suppressed *Rhizoctonia* disease development in the early stages of plant growth (4 weeks postplanting). A decrease in sprout mortality was observed in the plants grown from the treated tubers. In particular, sprout mortality in the control was 19.9%. Treatment with *M. robertsii* decreased plant mortality 3.15-fold compared to the control, but the difference was not significant (Dunn’s test, *P* = 0.129). At the same time, sprout mortality was not detected after treatment with *B. bassiana* (Dunn’s test, *P* = 0.031 compared to the control).

**Figure 4:**
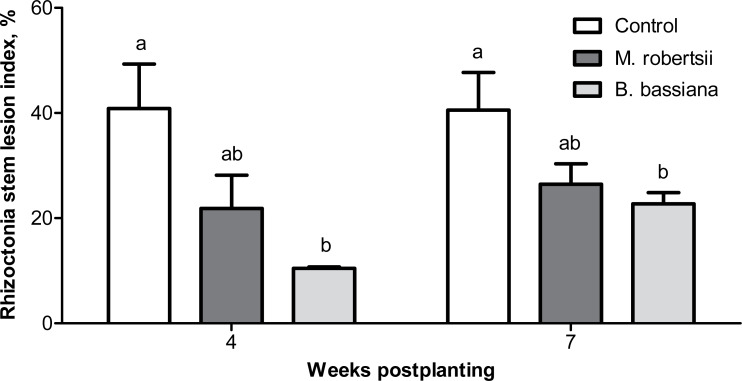
Effect of the treatment of seed tubers with entomopathogenic fungi on the damage to potato stems caused by *Rhizoctonia* disease during the growing season (2018). The bars indicate the standard error of the means (four biological replicates, five plants in each). The same letters indicate insignificant differences in the RSI between all treatments (Dunn’s test, Fisher’s LSD, *P* > 0.05).

Four weeks postplanting, we registered a trend of a decrease in the *Rhizoctonia* disease intensity (RSI) on the stems of plants treated with *M. robertsii* (1.87-fold compared to the control; Dunn’s test, *P* = 0.078, Fig. 4). A significant 3.61-fold decrease in the RSI was registered in plants treated with *B. bassiana* (Dunn’s test, *P* = 0.003 compared to the control). Seven weeks postplanting, a decrease in the RSI was also documented (Fig. 4). Similarly, the decrease after treatment with *M. robertsii* was marginal (Fisher’s LSD, *P* = 0.070 compared to the control), and a significant difference was detected after treatment with *B. bassiana* (Fisher’s LSD, *P* = 0.029 compared to the control).

**Figure 5:**
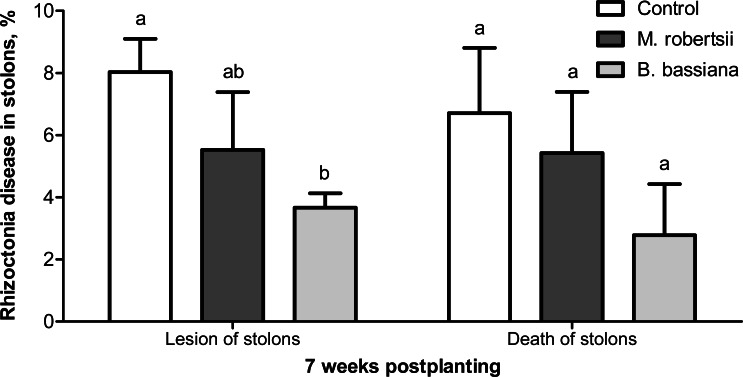
Effect of the treatment of seed tubers with entomopathogenic fungi on damage to potato stolons caused by *Rhizoctonia* disease (7 weeks postplanting). The bars indicate the standard error of the means (four biological replicates, five plants in each). The same letters indicate insignificant differences in damage to potato stolons between all treatments (Dunn’s test, Fisher’s LSD, *P* > 0.05).

At the 7-week postplanting, we registered a decreased number of damaged stolons in the plants treated with *B. bassiana* (2.2-fold compared to the control, Fisher’s LSD, *P* = 0.029, Fig. 5). Treatment with *M. robertsii* led to a 1.4-fold decrease in the number of damaged stolons, but the difference was marginal (Fisher’s LSD, *P* = 0.070 compared to the control). Treatment with fungi led to 1.2- and 2.4-fold decreases in the number of dead stolons after treatment with *M. robertsii* and *B. bassiana*, respectively, but these differences were not significant (Dunn’s test, *P* ≥ 0.141 compared to the control, Fig. 5).

The analysis of the new crop tubers showed that entomopathogenic fungi suppressed sclerotial forms of *Rhizoctonia* disease (Table 2). The proportion of healthy tubers (free from all *Rhizoctonia* disease symptoms) after treatment with the entomopathogenic fungi was not significantly different from that in the control (Fisher’s LSD, *P* ≥ 0.075, Table 2). However, the proportion of tubers free from sclerotia significantly increased after treatment with *B. bassiana* and *M. robertsii* (Fisher’s LSD, *P* < 0.016 compared to the control). The sclerotium index was decreased 1.7-2-fold (Fisher’s LSD, *P* = 0.016 compared to the control, Table 2). The biological efficacy of the entomopathogenic fungi toward *Rhizoctonia* disease was high during all potato ontogeny stages, ranging from 40–100% (Table 3).

### Influence of fungi on potato growth and the tuber crop

**Figure 6:**
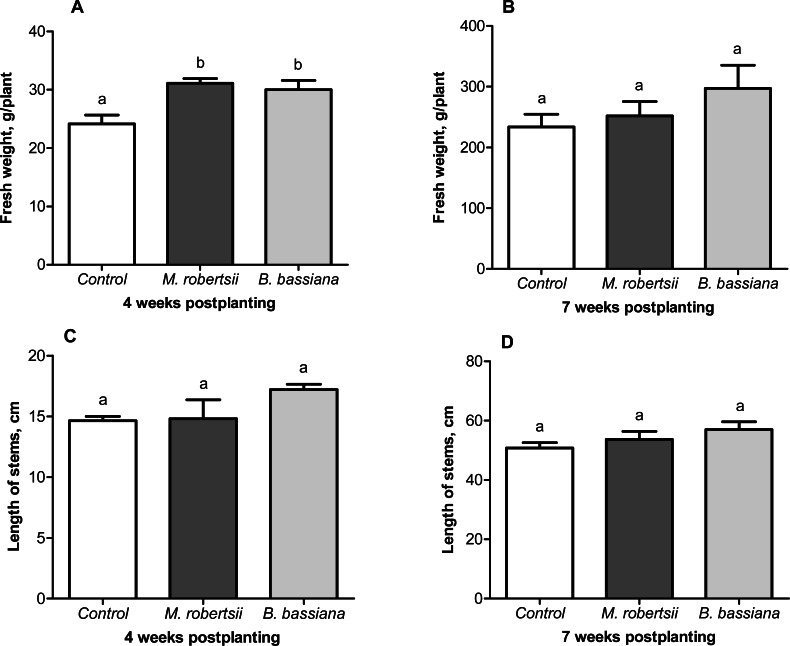
Effect of the treatment of seed tubers with entomopathogenic fungi on plant growth. The bars indicate the standard error of the means (four biological replicates, five plants in each). The same letters indicate insignificant differences in the length of stems and wet weight between all treatments (Fisher’s LSD, *P* > 0.05).

A growth-promoting effect of the entomopathogenic fungi on the potato plants was revealed. At 4 weeks postplanting, we observed an increase in plant weight after treatment with entomopathogenic fungi (Fig. 6A). Treatment with *M. robertsii* promoted a significant increase in plant fresh weight (6.96 g/plant; Fisher’s LSD, *P* = 0.037 compared to the control). At the same time, *B. bassiana* induced an increase of 5.85 g/plant, and the significance of this difference was at the marginal level (Fisher’s LSD, *P* = 0.070 compared to the control). Plant length after fungal treatment was only slightly increased compared to the control (0.18–2.56 cm) (Fisher’s LSD, *P* ≥ 0.091, Fig. 6C).

**Table 2:** The effect of entomopathogenic fungi on the forms of *Rhizoctonia* disease in tubers (mean ± SE, 120 tubers from each treatment).

**Forms of *Rhizoctonia* disease**	**Share of tubers, %**		
	**Control**	***M. robertsii***	***B. bassiana***
No signs of blight	**42.50 ± 4.77**^ab^	**53.75 ± 4.27**^a^	**36.25 ± 2.39**^b^
Weakly scab-like symptoms	20.00 ± 5.77	8.75 ± 2.39	25.00 ± 4.56
Scab-like symptoms	10.00 ± 2.50	13.33 ± 3.82	10.00 ± 3.54
Dry core symptoms	1.25 ± 1.25	–	–
Scab-like symptoms + cracks	15.00 ± 7.07	23.75 ± 3.75	25.00 ± 5,40
**Without black scrub**	**86.25 ± 2.39**^a^	**96.25 ± 2.39**^b^	**96.25 ± 2.39**^b^
*Black scrub (the surface of tuber covered with sclerotia)*			
On 1/2	–	–	–
1/4	–	–	–
1/10	6.25 ± 1.25	–	–
Single sclerotia	3.75 ± 1.25	1.25 ± 1.25	2.50 ± 1.44
*Combined forms*			
Sclerotia 1/2 + scab-like	–	–	–
Sclerotia 1/4 + scab-like	1.25 ± 1.25	–	–
Sclerotia 1/10 + scab-like	1.25 ± 1.25	1.25 ± 1.25	–
Single sclerotia + scab-like	1.25 ± 1.25	–	–
Sclerotia 1/2 + scab-like + cracks	–	–	–
Sclerotia 1/4 + scab-like + cracks	–	–	–
Sclerotia 1/10 + scab-like + cracks	–	–	–
Single sclerotia + scab-like + cracks	–	1.25 ± 1.25	–
***Sclerotium index***	**0.86 ± 0.07**^a^	**0.44 ± 0.09**^b^	**0.50 ± 0.10**^b^

**Notes.**

The same letters indicate insignificant differences between treatments (Fishers LSD, *P* > 0.05).

**Table 3:** Efficiency of the application of entomopathogenic fungi for tuber treatment.

**Treatment**	**Biological efficiency, % on**			
	**Sprouts**	**Stems**	**Stolons**	**Tubers**
*M. robertsii*	68.3	45.8	30.9	48.8
*B. bassiana*	100	73.1	54.3	40.7

At the following time-point (7 weeks postplanting), significant differences between the control and treated plants were not registered for either plant weight (Fisher’s LSD, *P* ≥ 0.149, Fig. 6B) or length (Fisher’s LSD, *P* ≥ 0.098, Fig. 6D).

Treatment with the entomopathogenic fungi did not significantly affect the number of stems per potato plant, the number of stolons per potato plant or the number of tubers per potato plant at either time-point (data dot shown). Treatment with the fungi slightly increased the tuber crop, but the differences were not significant (Fisher’s LSD, *P* ≥ 0.096, Table 4). Nevertheless, the crop return resulting from *M. robertsii* treatment was 7.0 kg per 100 potato plants, and that resulting from *B. bassiana* treatment was 11.1 kg per 100 potato plants, or 12.1 and 19.2%, respectively. The proportion of the medium-sized fraction was increased after treatment with *M. robertsii,* and the proportion of the large fraction was increased after treatment with *B. bassiana,* but the differences were insignificant compared to the control (Table 4, see also ESM Fig. S1).

**Table 4:** Effect of entomopathogenic fungi on the fractional composition and yield of potato following the treatment of seed tubers (mean ± SE, 4 biological replicates, 10 plants in each).

**Treatment**	**Fractional composition, %**			**Biological yield/100 plants**		
	**Small fraction**	**Medium fraction**	**Large fraction**	**kg**	**Yield increase**	
					**kg**	**%**
Control	4.83 ± 0.48^a^	41.33 ± 2.81^a^	53.85 ± 2.98^ab^	57.73 ± 4.60^a^	–	–
*M. robertsii*	6.03 ± 0.23^a^	43.65 ± 5.97^a^	50.33 ± 6.07^a^	64.70 ± 3.02^a^	7.0	12.1
*B. bassiana*	3.25 ± 0.38^b^	31.55 ± 2.29^a^	65.20 ± 2.18^b^	68.85 ± 4.83^a^	11.1	19.2

**Notes.**

The same letters indicate insignificant differences between treatments (Fisher’s LSD, *P* > 0.05).

### CFUs in soil and colonization of plants

#### Soil CFUs

The soil plated before the start of the experiment showed a low frequency of the entomopathogenic fungi: *B. bassiana*—133.33 ± 57.38 CFUs/g dry soil, and *M. robertsii*—155.56 ± 75.90 CFUs/g dry soil. At the end of the vegetation period (13 weeks postplanting), the average CFUs of the entomopathogenic fungi in the rhizosphere soil of the treated plants were 3977.78 ± 965.84 CFUs/g dry soil for *M. robertsii* and 2955.56 ± 718.82 CFU/ g dry soil for *B. bassiana.* In the control samples of rhizosphere soil, entomopathogenic fungi were detected at the following rates: *B. bassiana*—88.89 ± 36.29 CFUs/g dry soil, and *M. robertsii*—266.67 ± 120.36 CFUs/g dry soil.

#### Plant colonization

At 4 weeks postplanting, we did not observe colonization of potato plant tissues (leaves, stems and roots) by *M. robertsii* or *B. bassiana*. Notably, roots plated without sterilization (washed solely with the water-Tween solution) showed ample surface contamination by the experimental fungi in 70–80% of the plants (ESM Fig. S2), which suggests active fungal development in the rhizosphere zone. In the control samples of the plants, colonies of the entomopathogenic fungi were not detected.

At 7 weeks postplanting, the percentage of the plants exhibiting systemic colonization by the fungi (leaves, stems and roots) was low (*M. robertsii*—5.26%, *B. bassiana*—5.88%), and the difference between these treatments was not significant (Fisher’s exact test, *P* = 0.730). The rates of local colonization of the roots were 31.58% for *M. robertsii* and 47.06% for *B. bassiana* (Fisher’s exact test, *P* = 0.377). The entomopathogenic fungi were not isolated from the control plants.

### Activity of POX

**Figure 7:**
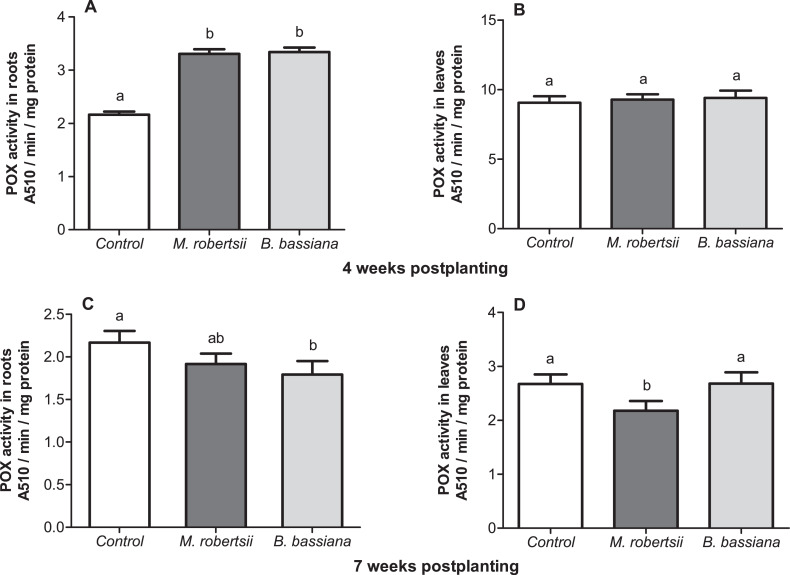
Effect of the treatment of seed tubers with entomopathogenic fungi on the levels of peroxidase activity in potato roots (A, C) and leaves (B, D). The bars indicate the standard error of the means (*n* = 20). The same letters indicate insignificant differences in POX activity in plants between treatments (Dunn’s test, *P* > 0.05).

At 4 weeks postplanting, we revealed a significant 1.5-fold increase in POX activity in the roots under the influence of both fungi (Dunn’s *P* ≤ 0.014, Fig. 7A). At the same time-point, enzyme activity in the leaves was close to the control level (Dunn’s test, *P* ≥ 0.687, Fig. 7B). At 7 weeks postplanting, POX activity in the roots tend to decrease in the plants that were treated with fungi (Fig. 7C). Treatment with *B. bassiana* led to a significant decrease compared to the control (Dunn’s test, *P* = 0.023 compared to the control), but treatment with *M. robertsii* led to insignificant differences (*P* = 0.162). POX activity in the leaves was not significantly changed in *B. bassiana-* treated plants (Dunn’s test, *P* = 0.949, Fig. 7D), but this parameter was significantly decreased after treatment with *M. robertsii* (Dunn’s test, *P* = 0.041 compared to the control).

## Discussion

This work demonstrated that the treatment of potato seed tubers with entomopathogenic fungi, followed by growth in open-ground conditions increased plant resistance to *Rhizoctonia* disease. We found for the first time that plants treated with the investigated strain of *B. bassiana* gained resistance to the pathogen during the vegetation period under the continental conditions of Western Siberia. In addition, plants treated with the fungus exhibited more rapid growth and increased tuber quality. Similar trends were observed after the treatment of seed tubers with the strain of *M. robertsii,* but these effects were weaker. We suggest that entomopathogens influence *R. solani* directly (as an antagonist) or indirectly (through the activation of the plant defense systems).

The results obtained in the dual fungal growth experiments showed antagonistic properties of *M. robertsii* and *B. bassiana* toward the phytopathogen *R. solani*. The activity of *Beauveria* and *Metarhizium* species against various phytopathogens has been described in previous studies on the interaction between fungi in vitro (Renwick, Campbell & Coe, 1991; Veselý & Koubová, 1994; Bark et al., 1996; Reisenzein & Tiefenbrunner, 1997; Lee et al., 1999; Sasan & Bidochka, 2013; Shternshis et al., 2014; Gothandapani et al., 2015; Barra-Bucarei et al., 2020). Many authors suggest a key role of entomopathogenic fungal metabolites in inhibition of phytopathagens. These suggestions have been confirmed in experiments involving cell-free culture filtrates (Renwick, Campbell & Coe, 1991; Bark et al., 1996; Sasan & Bidochka, 2013; Lozano-Tovar et al., 2017). It was also shown that *B. bassiana* produces an antifungal peptide (BbAFP1) that inhibits the growth of *Alternaria brassicae* (Tong et al., 2020). The activity of *Metarhizium* extracts against *Phytophthora sojae* and *Aphanomyces cochlioides* was associated with the secondary metabolites aurovertins and fungerin, N-(methyl-3-oxodec-6-enoyl)-2-pyrroline, and N-(methyl-3-oxodecanoyl)-2-pyrroline (Putri et al., 2014).

It should be noted that the data on the influence of the entomopathogenic fungi on *R. solani* are very contradictory. Lee et al. (1999) and Shternshis et al. (2014) observed growth inhibition of *R. solani* during cocultivation with entomopathogenic fungi, while Veselý & Koubová (1994) showed that *Beauvaria* spp. did not exhibit any antagonistic activity against *R. solani*. Guo et al. (2005) demonstrated inhibitory activity of the metabolic peptide isapheline from the *Isaria feline* mycelium on mycelial growth in *R. solani*. However, Kang et al. (2018) did not observe radial growth inhibition of *R. solani* by *Isaria javanica.* We also did not observe inhibition of radial colony growth of this phytopathogen by investigated strains of *M. robertsii* and *B. bassiana*. Nonetheless, the long-term cocultivation of *M. robertsii* and *B. bassiana* with *R. solani* allowed us to observe the braiding of *R. solani* hyphae by entomopathogens with aerial mycelium necrotization and suppression of sclerotium formation.

Many authors have reported that endophytic entomopathogenic fungi decrease plant damage caused by various phytopathogens (reviwed by Bamisile et al., 2018; Jaber & Ownley, 2018; Vega, 2018), including *R. solani* (Griffin et al., 2005; Ownley, Dee & Gwinn, 2008; Arab & El-Deeb, 2012). We found for the first time that the treatment of potato tubers with fungi before planting led to a decrease in *Rhizoctonia* disease symptoms throughout the growing season. In particular, it decreased the damage to sprouts, stems and stolons as well as sclerotium formation on the tubers of new crops. Interestingly, *M. robertsii* was more active against *R. solani* than *B. bassiana* in vitro, but in field conditions, we obtained the opposite results, at least with respect to *Rhizoctonia* disease on the sprouts, stems and stolons. On the other hand, equal levels of the suppression of sclerotium index on tubers of the new crop was observed after the *M. robertsii* and *B. bassiana* treatments. These effects may be observed because members of the genus *Metarhizium* mostly colonize rhizosphere and internal root tissues (Hu & St Leger, 2002; St Leger, 2008; Steinwender et al., 2015; Barelli, Moreira & Bidochka, 2018), while *Beauveria* species adapted to colonize different plant tissues (roots, stems and leaves) (Behie, Jones & Bidochka, 2015). It is likely that the differences in *Rhizoctonia* inhibition are linked to these adaptations. Future studies could focus on the quantification of the colonization of potato organs by the investigated fungi using molecular tools.

It should be noted that the inoculation of tubers with entomopathogenic fungi is a more technologically challenging strategy compared to soil drenching or spraying. Preplanting treatment allows the observation of the effects on the pathogen directly at the time of its activation in the early stages of plant ontogenesis. However, the drenching of soil with a *M. robertsii* conidial suspension also led to the suppression of potato *Rhizoctonia* disease in the field conditions of Western Siberia (ESM Fig. S3).

It is important that an optimal planting density is employed as this affects the basic structure of the potato crop in the early stage (during the 4 weeks postplanting). During this period, we observed decreases in sprout mortality and the *Rhizoctonia* stem lesion index as well as an increase in plant weight. A growth stimulation effect on plants after treatment with *Metarhizium* and *Beauveria* fungi has been registered by many other researchers in various plants (Gomez-Vidal et al., 2009; Liao et al., 2014; Lopez & Sword, 2015; Gathage et al., 2016; Jaber & Enkerli, 2016; Jaber & Enkerli, 2017; Jaber & Araj, 2017; Raad et al., 2019), including potato (Krell et al., 2018). This phenomenon may have several potential causes, such as (1) the additional supply of nitrogen via the mycelium to plant roots (Behie, Zelisko & Bidochka, 2012; Behie & Bidochka, 2014); (2) the inducible production of regulatory proteins, altering metabolic activity in the plants (Gomez-Vidal et al., 2009); (3) the activation of phytohormone production (Raad et al., 2019); and (4) the synthesis of hormone-like substances by the fungi themselves (for example, indole-3-acetic acid) (Liao et al., 2017). Nevertheless, most of these studies were performed in sterile soils or under greenhouse conditions. We are the first to show a plant-growth promoting effect under the field conditions of Western Siberia, which is in a zone of risky agriculture.

We documented an increase in POX activity after treatment with both strains of fungi at 4 weeks postplanting. We suggest that the colonization of the soil and rhizosphere zone of potato led to an increase in peroxidase activity in the roots. The activation of plant-protective enzymes, particularly peroxidase, polyphenol oxidase and chitinase, has been observed in studies considering the interactions between plants and antagonistic bacteria (*Pseudomonas fluorescens*, *Bacillus subtilis*) and between plants and entomopathogenic fungi (Karthiba et al., 2010; Senthilraja et al., 2013; Prabhukarthikeyan, Saravanakumar & Raguchander, 2014; Prabhukarthikeyan et al., 2017). Independent endophytic colonization with entomopathogenic fungi may induce plant resistance to phytopathogens and lead to an increase in the activity of protective compounds (Hirano et al., 2008; Maksimov et al., 2014; Maksimov et al., 2015). Raad et al. (2019) assessed *Arabidopsis thaliana* transcriptomic and metabolic responses to *B. bassiana* colonization and the resistance of the plants to the phytopathogen *Sclerotinia sclerotiorum.* These authors revealed the upregulation of genes related to the synthesis of components of the phytoalexin, jasmonic, and salicylic acid signaling pathways.

Active vegetative mass synthesis, stolon formation and tuber setting are characteristic of the bud-formation and flowering periods (7 weeks postplanting). During this period, we also registered a decrease in lesions on the stems and stolons caused by *Rhizoctonia* disease. However, at this experimental time-point, we did not observe significant differences in growth parameters of POX activity between the treated and control plants or exception of slight decrease in POX activity under the influence of *M. robertsii*. Thus, the strongest growth-related effects and immune reactions in the plants were observed during earlier stages of the interaction between plants and fungi. Further research could be focused on the dynamics of the immune reactions and growth parameters of potatoes after treatment with entomopathogenic fungi.

At the final stage of the experiment (13 weeks postplanting), when the tubers of the new crop had finally formed, we registered only a trend of an increase in the total tuber weight (12.1–19.2%), but tuber quality (the decrease of *Rhizoctonia* sclerotia on the surface of tubers) was significantly improved. It is important to note that the sclerotium index of the tubers was significantly lower than that of the control by 1.7-2-fold. The low level of tuber infection means that these tubers can be used as seed tubers because sclerotia are the main source of Rhizoctonia infection (Banville, Carling & Otrysko, 1996; Tsror, Barak & Sneh, 2001; Tsror & Peretz-Alon, 2005; Daami-Remadi, Zammouri & Mahjoub, 2008). It should be notice that the soil in the root area of the treated plants was highly contaminated with both *M. robertsii* and *B. bassiana* compared to the control plants, which suggested the fungi remained and proliferated in the rhizosphere zone (Inglis et al., 1997; Hu & St Leger, 2002) and might interact with *R. solani* during vegetation season.

## Conclusions

This work is the first study of the effect of entomopathogenic fungi on potato cultivation under the continental climate of Siberia. The study showed that the preplanting treatment of seed tubers with the conidia of entomopathogenic fungi protected against *Rhizoctonia* disease during the vegetative growth. In particular, the treatment decreased the development of the disease on the stems and stolons of potato plants and decreased the formation of *R. solani* sclerotia on the surface of new crop tubers. The suppression of *Rhizoctonia* disease could be caused by both the antagonistic properties of these fungi and the activation of the host-plant resistance response. Further studies should focus on the screening of strains for the inhibition of *Rhizoctonia* disease on potato under the conditions of Western Siberia as well as the development of different formulations for potato treatments.

##  Supplemental Information

10.7717/peerj.9895/supp-1 Figure S1 Phenotypes of potato plants that were treated and untreated with entomopathogenic fungi

10.7717/peerj.9895/supp-2 Figure S2 The growth of entomopathogenic fungi from the surface of unsterilized potato roots (washed three times with a water-Tween solution) A—*B. bassiana*; B—*M. robertsii*.

10.7717/peerj.9895/supp-3 Figure S3 Effect of soil drenching with a *Metarhizium robertsii* conidia suspension in period of potato sprout formation (2.5 × 10^7^, 0.5 L/plant) on damage to the stems and stolons of potato caused by *Rhizoctonia* disease during the growing seas The bars indicate the standard error of the mean (4 biological replicates, 5 plants in each). The same letters indicate insignificant differences in the damage to potato stems and stolons between all treatments (Fisher’s LSD, *P* > 0.05).

10.7717/peerj.9895/supp-4 Dataset S1 The raw data of the plant colonization by entomopathogenic fungi and their CFUs in soil, parameters of *Rhizictonia* infection, potato plant growth (height and weight), tuber crop, antagonistic activity and peroxidase activity

## Additional Information and Declarations

### Data Availability

The following information was supplied regarding data availability: The raw data of the plant colonization by entomopathogenic fungi and their CFUs in soil, parameters of Rhizictonia infection, potato plant growth (height and weight), tuber crop, antagonistic activity and peroxidase activity are available in the Supplemental Files.
