# Supplementary figures and images for: Entomopathogenic fungi decrease Rhizoctonia disease in potato in field conditions
Source: PeerJ. 2020 Sep 16;8:e9895. doi: 10.7717/peerj.9895 (PMC7501787; doi:10.7717/peerj.9895)

7 weeks postplanting

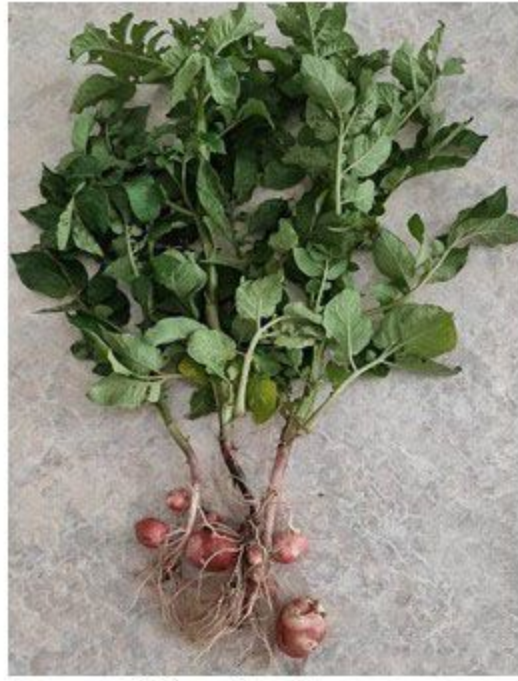

**Control**

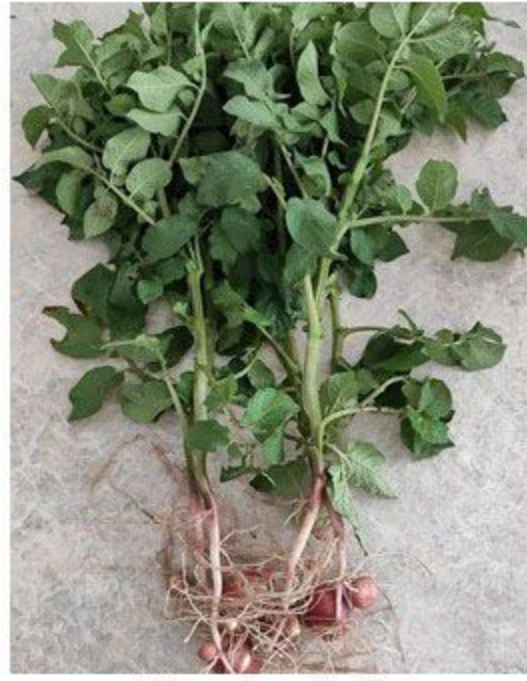

***M. robertsii***

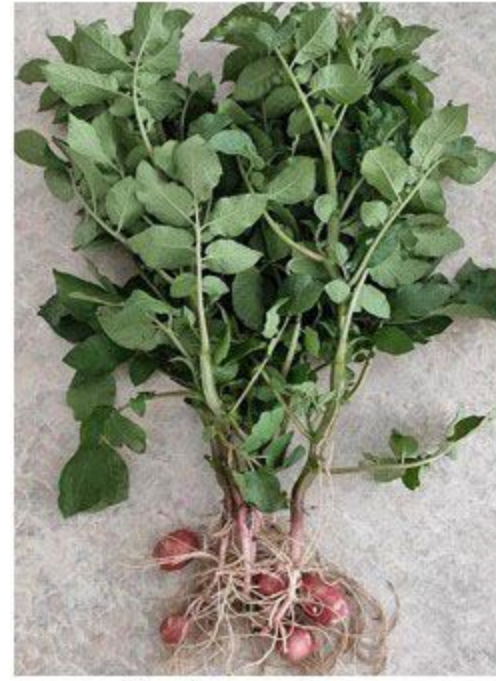

***B. bassiana***

13 weeks postplanting

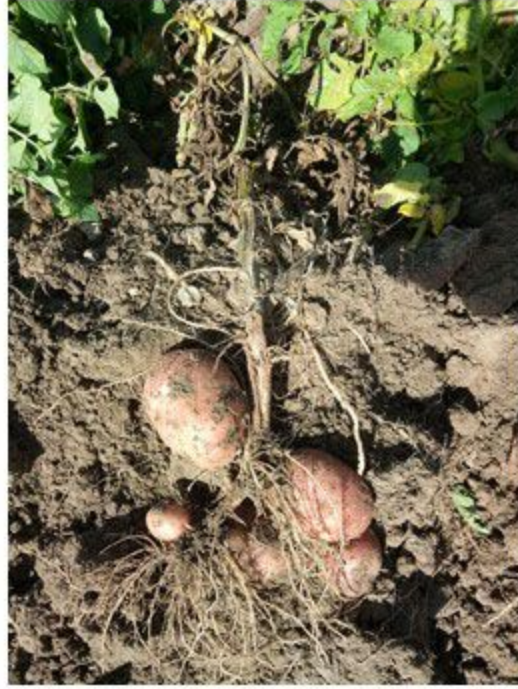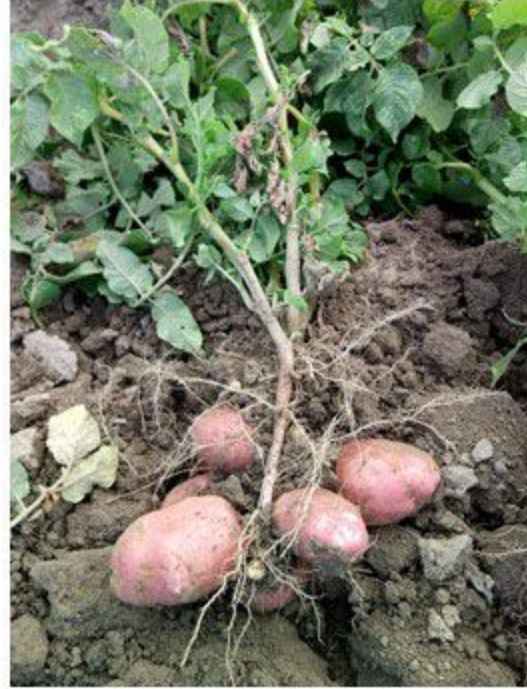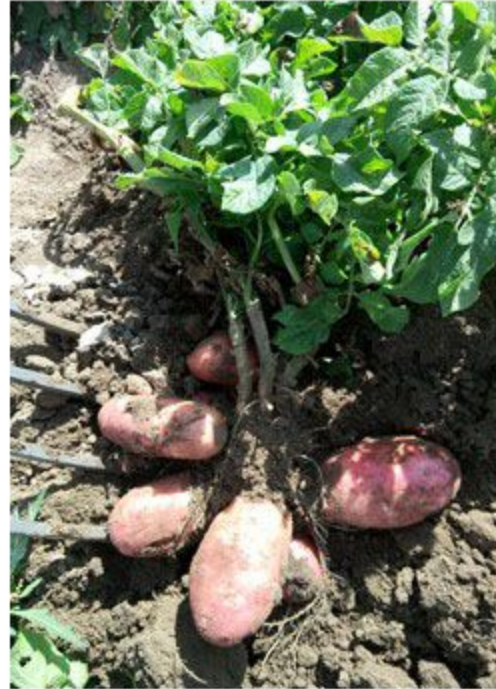

Supplement: Figure S1 [file peerj-08-9895-s001.pdf]

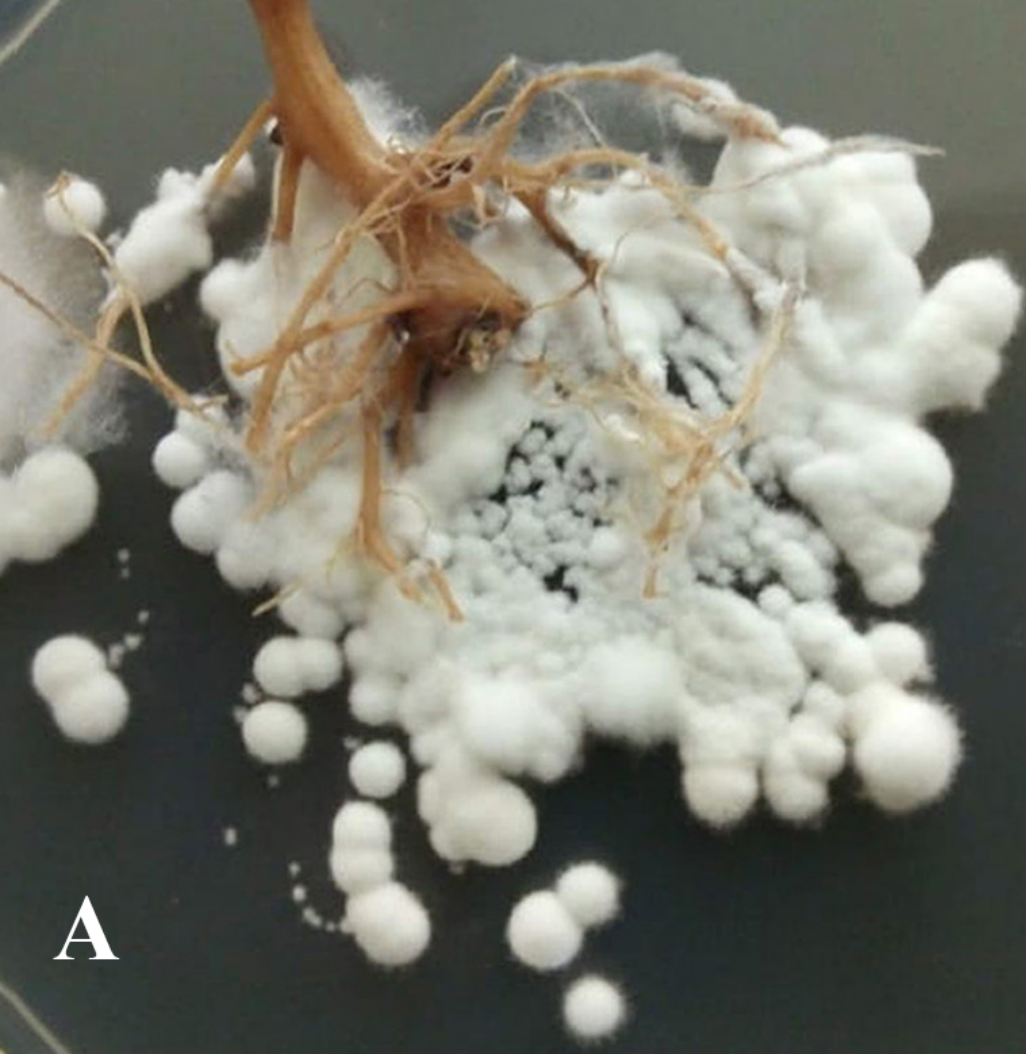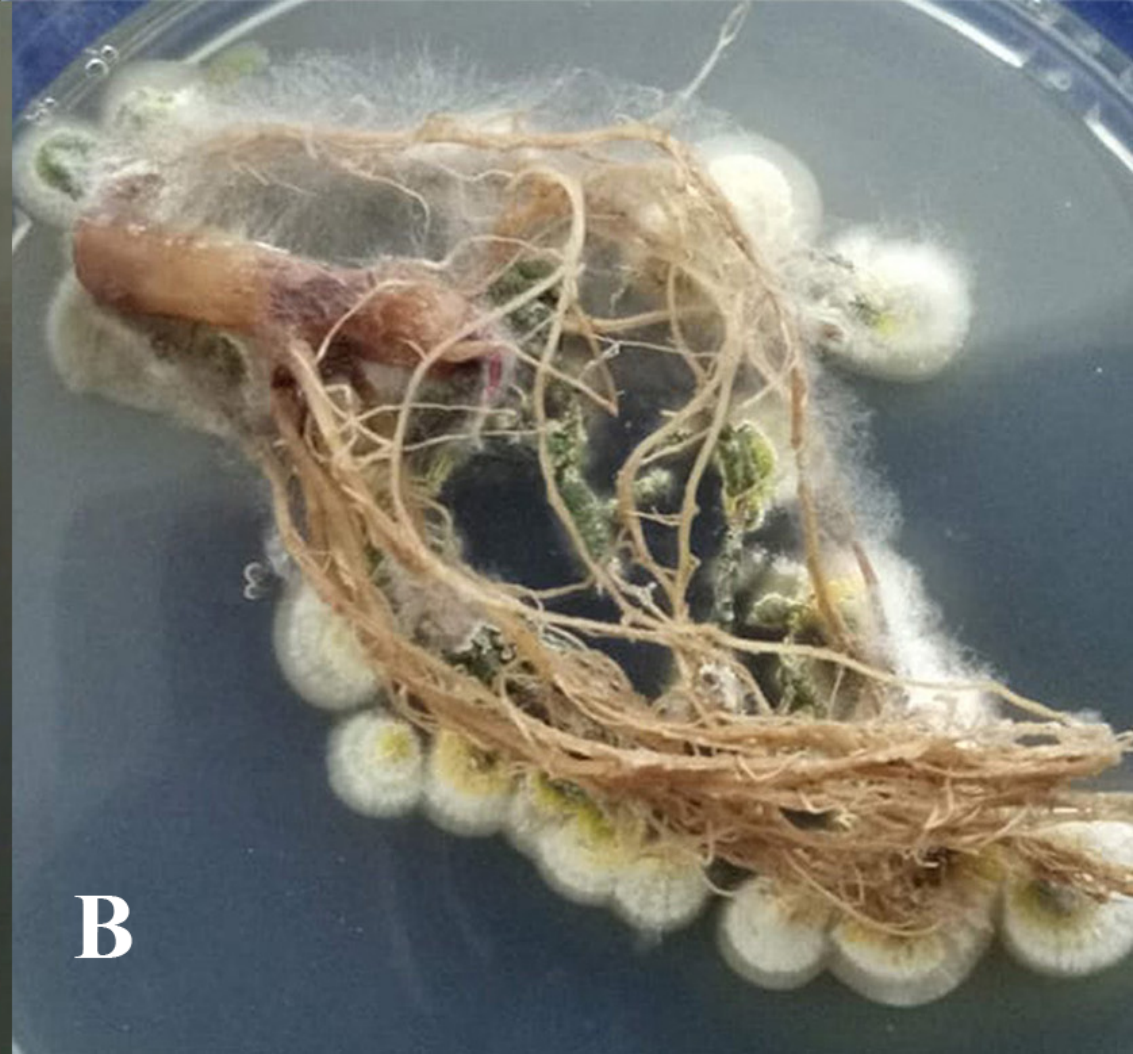

Supplement: Figure S2 — A—B. bassiana; B—M. robertsii. [file peerj-08-9895-s002.pdf]

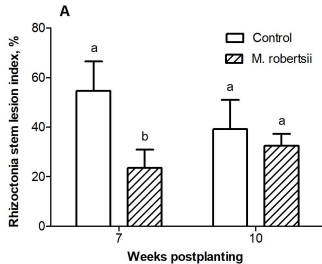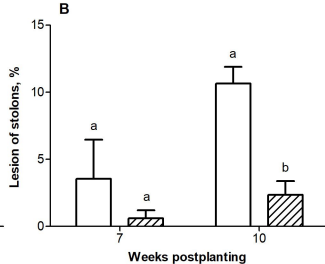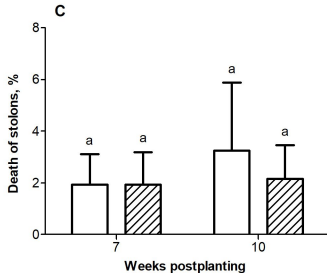

Supplement: Figure S3 — The bars indicate the standard error of the mean (4 biological replicates, 5 plants in each). The same letters indicate insignificant differences in the damage to potato stems and stolons between all treatments (Fisher’s LSD, P > 0.05). [file peerj-08-9895-s003.pdf]
